# Human Papillomavirus Testing and Size of CIN3: Implications for the Risk of Microinvasive Cervical Carcinoma

**DOI:** 10.3390/cancers18030396

**Published:** 2026-01-27

**Authors:** Mario Preti, Annibale Biggeri, Guglielmo Ronco, Maria Kyrgiou, Raffaella Rizzolo, Paola Armaroli, Niccolò Gallio, Murat Gultekin, Federica Zamagni, Silvano Costa, Pedro Vieira-Baptista, Fulvio Borella, Stefano Cosma, Luigia Macrì, Christine Bergeron, Silvia Mancini, Laura De Marco, Daniele Tota, Lauro Bucchi

**Affiliations:** 1 Department of Surgical Sciences, St. Anna University Hospital, Via Ventimiglia 3, 10126 Torino, Italy; mario.preti@unito.it (M.P.); niccolo.gallio@edu.unito.it (N.G.); fulvio.borella@unito.it (F.B.); stefano.cosma@unito.it (S.C.); 2 Unit of Biostatistics, Epidemiology and Public Health, Department of Cardiac, Thoracic, Vascular Sciences and Public Health, University of Padua, Via Leonardo Loredan 18, 35131 Padua, Italy; annibale.biggeri@ubep.unipd.it; 3 Città della Salute e della Scienza University Hospital, Corso Bramante 88, 10126 Turin, Italy; guglielmo.ronco@cpo.it (G.R.); raffaella.rizzolo@cpo.it (R.R.); 4 Institute of Reproductive and Developmental Biology, Department of Metabolism, Digestion and Reproduction—Surgery and Cancer, Faculty of Medicine, Imperial College London, Du Cane Road, London W12 0NN, UK; m.kyrgiou@imperial.ac.uk; 5 Imperial College Healthcare NHS Trust, Du Cane Road, London W12 0HS, UK; 6 Epidemiology and Screening Unit, Reference Centre for Epidemiology and Cancer Prevention (CPO), Città della Salute e della Scienza University Hospital, Via Cavour 31, 10123 Turin, Italy; paola.armaroli@cpo.it; 7 Division of Gynaecological Oncology, Department of Obstetrics and Gynaecology, Hacettepe University, Faculty of Medicine, Sihhiye, Ankara 06100, Türkiye; mrtgultekin@yahoo.com; 8 Emilia-Romagna Cancer Registry, Romagna Cancer Institute, IRCCS Istituto Romagnolo per lo Studio dei Tumori (IRST) Dino Amadori, Via Piero Maroncelli 40, 47014 Meldola, Italy; silvia.mancini@irst.emr.it (S.M.); lauro.bucchi@irst.emr.it (L.B.); 9 Gynaecology Division, Villa Chiara Hospital, Via Porrettana 170, 40033 Casalecchio di Reno, Italy; costa.silvano@libero.it; 10 Department of Gynecology-Obstetrics and Pediatrics, Faculdade de Medicina da Universidade do Porto, Alameda Professor Hernâni Monteiro, 4200-319 Porto, Portugal; pedrovieirabaptista@gmail.com; 11 HPV and Vulvovaginal Pathology Unit, Hospital Lusíadas Porto, Av. da Boavista 171, 4050-115 Porto, Portugal; 12 Pathology Unit, Città della Salute e della Scienza University Hospital, Corso Bramante 88, 10126 Turin, Italy; lmacri@cittadellasalute.to.it (L.M.); datota@cittadellasalute.to.it (D.T.); 13 CerbaPath, 30-32 Boulevard de Vaugirard, 75015 Paris, France; bergeron@lab-cerba.com; 14 Cancer Epidemiology Unit, Reference Centre for Epidemiology and Cancer Prevention (CPO), Città della Salute e della Scienza University Hospital, Corso Bramante 88, 10126 Turin, Italy; laura.demarco@cpo.it

**Keywords:** cervical cancer screening, human papillomavirus test, cervical intraepithelial neoplasia, lesion size, linear extension, glandular crypt involvement, stromal microinvasion

## Abstract

(1) Background/Objectives: Human papillomavirus (HPV) testing is hypothesised to detect cervical intraepithelial neoplasia grade 3 (CIN3) earlier than cervical cytology, which could translate into several clinical benefits. This study aimed to confirm that HPV testing detects CIN3 lesions of smaller size (or linear extension) and to assess whether this is associated with a decreased risk of stromal microinvasion (≤3 mm) (microinvasive or stage IA1 cervical carcinoma). (2) Methods: The study was conducted in a referral centre for cervical pathology in Italy. Eligible were 3744 patients aged 30–64 years who underwent local excision of the cervix between 1992 and 2021 and were diagnosed with CIN3, with or without microinvasion. Data were analysed using logistic and multinomial regression models. (3) Results: Overall, 1156 (30.9%) CIN3 cases were detected by the HPV test, and 2588 (69.1%) by cervical cytology. The lesion size was smaller in HPV test-detected CIN3 (median, 6 mm; interquartile range (IQR), 4–8 mm) than in cytology-detected CIN3 (median, 7 mm; IQR, 5–9 mm; *p* < 0.001). HPV test-detected CIN3 was over 50% less likely to have a size >6 mm combined with massive glandular crypt involvement. Stromal microinvasion occurred in 20/1156 (1.7%) HPV test-detected lesions versus 87/2588 (3.4%) cytology-detected lesions (*p* = 0.006), corresponding to an approximately 50% lower age-adjusted risk. The smaller size of HPV test-detected CIN3 and its lower degree of glandular crypt involvement interacted additively, rather than multiplicatively, in reducing the risk of stromal microinvasion. Over 46% of the association between detection mode and stromal microinvasion was explained by the size/involvement composite variable. (4) Conclusions: HPV testing detects CIN3 lesions of smaller size than cervical cytology. HPV test-detected CIN3 has a lower risk of stromal microinvasion. This association is mediated to a substantial extent by the smaller lesion size and the less extensive glandular crypt involvement, which interact in an additive manner. These findings may have other important clinical implications. First, the prevalence of disease persistence after treatment may decrease. Second, smaller lesions are likely to be treated with more limited excisions. Third, this may contribute to a lower rate of preterm birth in subsequent pregnancies.

## Simple Summary

This study was primarily aimed to confirm that human papillomavirus (HPV) testing detects cervical intraepithelial neoplasia grade 3 (CIN3) earlier than cervical cytology. In a group of 3744 CIN3 patients aged 30–64 years undergoing local excision of the cervix, we found that HPV testing detects CIN3 lesions of smaller size (or linear extension) and that these lesions have a lower risk of stromal microinvasion (microinvasive or stage IA1 cervical carcinoma). This association is mediated to a substantial extent by the smaller lesion size and the lower degree of glandular crypt involvement, which interact in an additive manner. Other potential clinical implications of a smaller lesion size include a decreased prevalence of disease persistence, the use of more limited excisions and, consequently, a lower rate of preterm birth in subsequent pregnancies.

## 1. Introduction

Cervical cancer is caused by persistent infection with oncogenic human papillomavirus (HPV) types, and the rationale for cervical cancer screening is to detect and treat high-grade precancerous lesions (cervical intraepithelial neoplasia grade 2–3, CIN2–3) before they progress to invasive cervical cancer. Compared with cervical cytology, HPV-based screening offers a higher detection rate of CIN2-3 at the initial screening round and a lower one at subsequent rounds [1]. This pattern is compatible with a temporal shift indicating that HPV testing detects the disease earlier than cervical cytology. Consequently, HPV-based screening is expected to identify high-grade CIN lesions in a more initial phase of growth and with a smaller size [2,3,4].

This may have important clinical consequences. The impact of screening on the incidence of invasive cervical cancer could be stronger, because previous studies have suggested that the smaller the high-grade CIN, the lower the risk of stromal invasion [5,6]. Furthermore, the prevalence of disease persistence after treatment is expected to decrease due to a reduced likelihood of involved resection margins [7,8]. Assuming that smaller lesions can be managed with more limited excisions, another expected effect is a reduction in the rate of preterm birth in subsequent pregnancies [9,10].

On the other hand, detecting smaller CIN lesions may also have undesirable effects, mainly related to the diagnostic process. First, the identification of smaller high-grade CIN lesions could adversely affect the accuracy of colposcopy [2,3,4] and the sensitivity of punch biopsy [2]. Second, the proportion of patients with histologic CIN on biopsy but no residual disease in the excision specimens is likely to increase, because smaller lesions have a higher probability of regression or of being completely removed with biopsy [11].

Despite their potential importance, most of these effects have been insufficiently confirmed. It also remains unclear whether the association between size and clinical outcome of CIN is modified by other characteristics of the disease, particularly glandular crypt involvement. The available literature is limited and largely descriptive [12,13,14], although both lesion size and crypt involvement are known to depend on histologic grade and Ki-67 expression [13].

We conducted a multiscope, multisetting clinical study on a large series of patients undergoing local excision of the cervix. In this article, we report the first analysis, which aimed (1) to confirm that the HPV test-detected CIN3 lesions have a smaller size than those detected by cervical cytology, and (2) to determine whether a smaller lesion size is associated with a lower risk of stromal microinvasion in the excision specimens, and how this association is influenced by the degree of glandular crypt involvement. The rationale for focusing on the risk of early stromal invasion is that, in cervical carcinomas with a depth of invasion exceeding 3 mm, the assessment of the intraepithelial component (i.e., CIN3) becomes challenging because increasing stromal invasion typically parallels a greater extent of superficial epithelial involvement.

## 2. Materials and Methods

### 2.1. Setting

The St. Anna University Hospital is a tertiary-level obstetrics and gynaecology centre serving the metropolitan area of Turin (northern Italy) and some neighbouring districts. With respect to cervical disease, women are referred for the diagnostic work-up of abnormal screening test results from the local organised screening programme as well as the opportunistic screening setting.

### 2.2. Screening Tests

The local organised screening programme, targeted at women aged 25–64 years, was introduced in 1992. Until March 2010, only cervical cytology was used. The samples were taken using a wooden Ayre’s spatula and a cytobrush and smeared on a slide. Since 2010, a plastic Ayre’s spatula and a cytobrush have been used. Cervical samples were collected in the PreservCyt solution (Thin Prep) and prepared with the Hologic 2000 or 5000 processor. Women with a diagnosis of atypical squamous cells of undetermined significance (ASC-US) (including a first-instance diagnosis) or more severe cytology were referred for colposcopic assessment.

For women aged 30–64 years, the programme switched progressively from cervical cytology- to HPV test-based screening between 2010 and 2018. Women aged 30–64 years eligible for a new screening round were randomised (cluster randomisation by year of birth) to cytology-based screening or HPV testing, with an increasing ratio of the latter to the former (Supplementary Figure S1). The Hybrid Capture 2 kit (Qiagen, Hilden, Germany) and the Anyplex II HPV28 Detection kit (Seegene Diagnostics, Seoul, Republic of Korea) were used. HPV test-positive women were triaged with cervical cytology. Aside from the transition to HPV testing, the screening protocol was not modified throughout the study period. The practice of cotesting was never used in this population.

The opportunistic screening practice, which is included in this study, takes place in private offices and is based on cervical cytology alone.

### 2.3. Colposcopy and Local Excision of the Cervix

At the St. Anna University Hospital, the protocol for colposcopy examination and local excision of the cervix during the study period (1992–2021) was the same for women from the organised screening setting and the opportunistic setting. The protocol included the application of 5% acetic acid and Lugol’s iodine staining. The colposcopes used were a Zeiss OPM1F (Karl Zeiss, Yena, Germany) and a Centrel Z3 (Centrel, Ponte San Nicolò, Padova, Italy). Small endocervical forceps were used to assess the visibility of the endocervical location of the squamocolumnar junction if needed.

Local excision of the cervix was performed under local anaesthesia using an electrosurgical generator in monopolar mode. The diathermy apparatus was set from 30 to 50 watts for cutting and from 30 to 60 watts for coagulation. Wire loop electrodes of 0.2 mm in diameter and 25 × 15, 25 × 10, 15 × 10 and 10 × 10 mm in width and height were used for cutting. In patients with very large lesions, 2–3 repeated ectocervical excisions were carried out or needle electrodes were used to tailor the resection. For lesions deeply extending into the endocervical canal, an additional apical specimen was obtained using a 10 × 10 or 15 × 15 mm wire loop electrode. After resection, the base of the wound was cauterised with a ball electrode using a pure coagulation frequency. The excised pieces were placed in a plastic tissue cassette and fixed in 10% buffered formaline.

### 2.4. Surgical Specimen Processing and Reporting

Cone and large loop excision of the transformation zone specimens were serially sectioned perpendicular to the transverse axis of the external os at 2–3 mm intervals, from the 9 o’clock to the 3 o’clock edge, according to published guidelines [15]. All slices were submitted sequentially for histologic examination and linear extension measurement.

During the study period, a total of five pathologists evaluated the specimens and reported the following cone details: anteroposterior dimension, transverse dimension, and length [16]; volume, estimated either as a half sphere or as a cone depending on the shape of the specimen; histologic diagnosis; linear extension of the lesion; degree of glandular crypt involvement (absent, partial (involvement of <50% of the gland depth), and massive); and status of the ectocervical, endocervical and deep margins. The pathologic examination for the linear extension and the degree of glandular crypt involvement has been conducted prospectively as part of routine pathology reporting since the early 1990s. The measurements were made in millimetres under microscopy using a micrometre in the 10× eyepiece. The micrometre had a linear scale of 10 mm divided in 100 intervals [17]. If the lesion involved more than one slide, linear extension was determined based on the number of consecutive slides involved, also taking the rare skip lesions into account.

The linear extension was defined as the maximum horizontal linear dimension of the lesion in millimetres, extending widely in the plane parallel to the eso- and endocervical mucosal surface [18]. The linear extension is the histopathologic characteristic of CIN3 we refer to as ‘lesion size’ in this article. This semantic choice is for reasons of clarity and consistency. Since the literature is very sparse on this subject, the use of the term ‘linear extension’ is uncommon and, moreover, is sometimes replaced with the variant term ‘linear extent’ [13,17,19,20].

### 2.5. Eligibility Criteria

**Figure 1:**
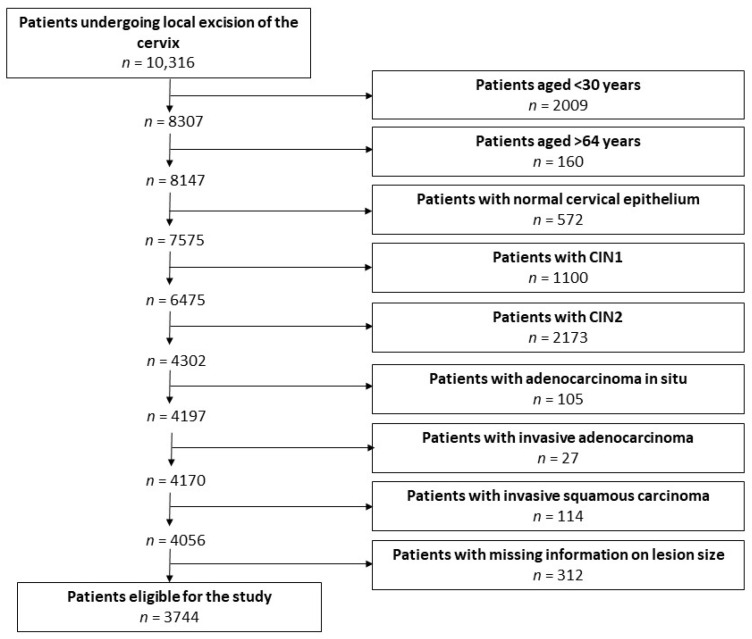
Flowchart of the process of selection of patients eligible for the study.

Trained medical personnel identified a consecutive series of 10,316 patients undergoing local excision of the cervix between 1992 and 2021 and retrieved their clinical data using a standard proforma. A flowchart illustrating the selection of patients eligible for this study is presented in Figure 1.

Included in the analysis were the patients who (1) were 30–64 years old and therefore within the age range for screening with either cervical cytology or HPV testing; (2) had a histologic diagnosis of CIN3, with or without stromal microinvasion, defined as an invasion (measured from the base of the epithelium from which the carcinoma arises to the deepest invasive focus) ≤3 mm (microinvasive or stage IA1 cervical carcinoma according to the International Federation of Gynaecology and Obstetrics 2018 staging system) [21]; and (3) had available information on the lesion size.

### 2.6. Statistical Methods

The size of CIN3 was treated as the main determinant of the risk of stromal microinvasion. Two observable variables were used to describe the overall magnitude of the lesion: the lesion size and the glandular crypt involvement. The lesion size was dichotomised at the median value (6 mm). Glandular crypt involvement was grouped as less than massive and massive. We then combined the lesion size and the glandular crypt involvement into a single composite 4-level variable, referred to as size/involvement (size ≤ 6 mm, involvement < massive; >6 mm, <massive; ≤6 mm, massive; >6 mm, massive).

Patient age and screening setting (organised, opportunistic) were treated as potential confounders or effect modifiers.

The detection mode (cervical cytology, HPV testing) was considered as another determinant of the main outcome, providing the rationale for studying the potential role of the size of CIN3 as a mediator of the association between detection mode and risk of stromal microinvasion.

The statistical analysis strategy was as follows. First, we evaluated the relationship between detection mode and size of CIN3, glandular crypt involvement and size/involvement composite variable. Second, we examined the association between detection mode and risk of stromal microinvasion. Third, we assessed the association between detection mode and stromal microinvasion after adjusting for the size/involvement variable using the so-called Baron and Kenny model [22,23]. Fourth, we ran a mediation analysis in order to estimate the percent of the association between detection mode and stromal microinvasion that can be explained by the size/involvement composite variable [24,25].

Then, in order to control for time and selection biases, we replicated the whole statistical analysis for the restricted period 2010–2018, when HPV testing and cervical cytology were randomly allocated.

Finally, we performed a sensitivity analysis excluding patients with CIN3 lesions with positive surgical margins, which were presumed to be incompletely excised and therefore to have an underestimated size.

All analyses were performed using the STATA statistical package, version 15.1 (Stata Corporation, College Station, TX, USA).

## 3. Results

### 3.1. Characteristics of Patients

Overall, 3744 eligible patients with CIN3 were identified, 2305 (61.6%) of whom were from the organised screening setting and 1439 (38.4%) from the opportunistic screening setting. There were 2588 (69.1%) patients detected by cervical cytology and 1156 (30.9%) detected by the HPV test.

### 3.2. Association Between Detection Mode and Size/Involvement

**Figure 2:**
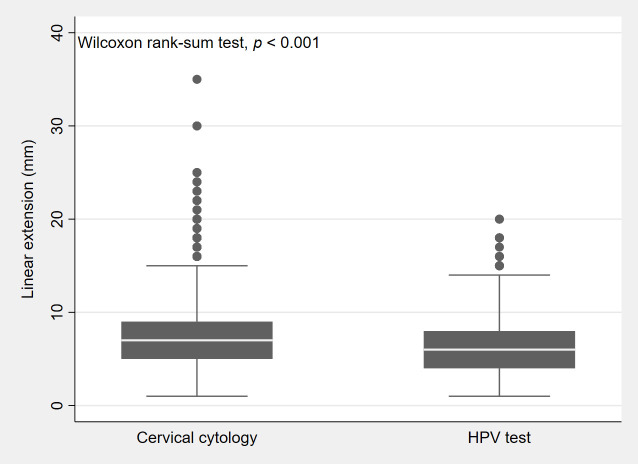
Box plot showing the distribution (25th, 50th, 75th percentile) by lesion size (or linear extension) of CIN3 lesions detected by cervical cytology and lesions detected by the HPV test.

Data in Figure 2 show that the size of CIN3 (overall median, 6 mm; interquartile range (IQR), 4–8 mm) was smaller in lesions detected by the HPV test (median, 6 mm; IQR, 4–8 mm) than in those detected by cervical cytology (median, 7 mm; IQR, 5–9 mm).

Table 1 shows the association of detection mode with a lesion size >6 mm and a massive glandular crypt involvement. Both estimates were adjusted for patient age. The detection by HPV testing was associated with an approximately one-third lower risk of CIN3 having a size >6 mm. We did not find any significant interaction between patient age and detection mode, whether patient age was entered as a categorical variable (likelihood-ratio (LR) test, *p* = 0.561) or a continuous variable (LR test, *p* = 0.463). Thus, patient age did not modify the effect of detection mode and was retained as a confounder in the model. As regards glandular crypt involvement, HPV test-detected CIN3 had an approximately 50% decreased risk of massive involvement. The regression analysis failed to find a significant interaction between patient age and detection mode (LR test, *p* = 0.863). Patient age was retained in the model as a confounder.

Table 2 shows that the detection mode was also significantly associated with the size/involvement variable. In particular, HPV test-detected CIN3 was over 50% less likely to have both a size >6 mm and a massive glandular crypt involvement.

### 3.3. Association Between Detection Mode and Presence of Stromal Microinvasion

Overall, stromal microinvasion was detected in 107/3744 (2.9%) patients. Table 3 shows that the prevalence of stromal microinvasion was 1.7% among lesions detected by the HPV test and 3.4% among those detected by cervical cytology (Pearson chi-square test, *p* = 0.006). After adjustment for patient age, the detection by HPV testing was confirmed to convey a 50% decrease in the risk of stromal microinvasion.

### 3.4. Association Between Size/Involvement and Presence of Stromal Microinvasion

The size of CIN3 was positively associated with the presence of stromal microinvasion, with an OR of 1.22 (95% confidence interval (CI): 1.17–1.26) for each millimetre increase in lesion size.

**Figure 3:**
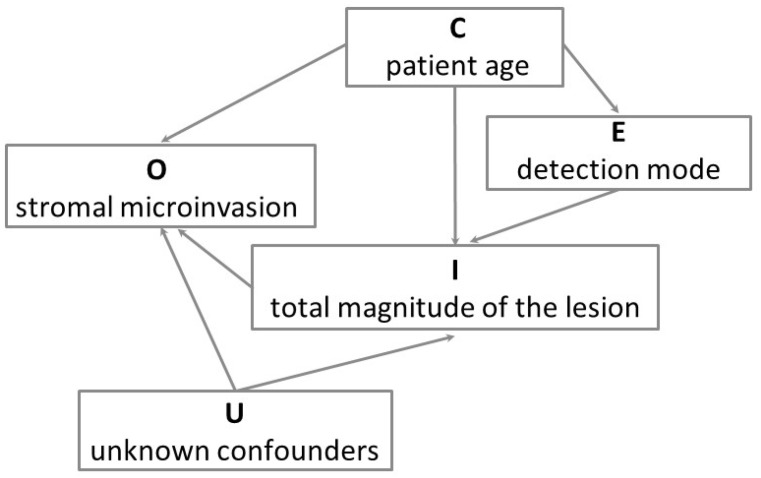
Directed acyclic graph of the model for the associations of patient age, detection mode and total magnitude of the lesion (i.e., the size/involvement composite variable combining the lesion size (or linear extension) and the degree of glandular crypt involvement) with the presence of stromal microinvasion in CIN3. O is the outcome variable (stromal microinvasion), E is the exposure variable (detection mode), I is the intermediate (mediator) variable (total magnitude of the lesion), C is the confounder variable (we only considered patient age), and U indicates the unknown confounders of the mediator–outcome association, which we assumed to be absent (conditional ignorability). This causal graph illustrates that the detection of CIN3 with HPV testing decreases the risk of stromal microinvasion through a reduction of the total magnitude of the lesion, that is, through a smaller lesion size and a lower degree of glandular crypt involvement.

Table 4 shows the results of a model including both the detection mode and the size/involvement variable. The detection mode was no longer a significant predictor of stromal microinvasion. By contrast, size/involvement showed a strong association with the risk of stromal microinvasion, indicating that the reduced risk observed for HPV test-detected CIN3 was mediated by its smaller size and its lower degree of glandular crypt involvement. When the lesion size was >6 mm and the glandular crypt involvement was massive, the OR for stromal microinvasion was 23.07. This value was only slightly higher than the sum of the individual effects (4.93 + 11.91 = 16.84) but far lower than their product (58.72), supporting an additive rather than multiplicative relationship between the two factors. Finally, no significant interaction was found between detection mode and size/involvement (LR test, *p* = 0.699). Figure 3 depicts the directed acyclic graph summarizing the results of this mediation analysis.

The mediation analysis showed that the share of the association between detection mode and stromal microinvasion that can be explained by the size/involvement composite variable was 46.4% (95% CI: 29.01–140.4).

Then, we replicated the whole statistical analysis for the restricted period 2010–2018, when HPV testing and cervical cytology were randomly allocated. The results (Supplementary Tables S1–S4) confirmed the findings of the primary analysis run on the full dataset.

A sensitivity analysis failed to demonstrate biases arising from the presence of CIN3 lesions with positive surgical margins (Supplementary Table S5).

## 4. Discussion

There are three key findings in this study. First, we confirmed the widely held but previously unproven hypothesis that the adoption of HPV testing in cervical screening leads to the detection of CIN3 lesions of smaller size than those detected by cervical cytology.

Second, we showed that HPV test-detected CIN3 is associated with a lower risk of stromal microinvasion (or microinvasive cervical carcinoma) compared with cytology-detected CIN3.

Third, using a formal mediation analysis, we demonstrated that this association is mediated to a substantial extent by the lesion size and the glandular crypt involvement. In other words, the smaller lesion size and the lower degree of glandular crypt involvement are the main factors through which the HPV test-based detection reduces the risk of stromal microinvasion. More specifically, the interaction between lesion size and glandular crypt involvement is additive rather than multiplicative. This suggests that an extensive glandular crypt involvement represents an additional quantitative burden of proliferating cells, and not a qualitatively distinct risk factor nor an independent marker of increased biologic aggressiveness.

A number of studies have addressed the association of colposcopic size of high-grade CIN with other characteristics of the disease, including, for example, risk of progression and regression [26], blood parameters [27], severity of the cytopathology and histopathology reports [28], high-grade residual disease after re-excision [29], pathologic upgrading or downgrading after conisation [30], and risk of recurrence [31]. By contrast, the lesion size and the degree of glandular crypt involvement have been largely neglected in studies on the natural history of cervical disease [32]. In particular, the interplay between the two features in determining the risk of progression has not previously been investigated. Kimura et al. have reported that they both depend upon histologic grade and Ki-67 expression [13]. A direct association between lesion size and risk of stromal microinvasion has so far been documented only in anecdotal studies published decades ago and not subsequently confirmed [5,6]. Massive glandular crypt involvement has mainly been linked to the risk of post-treatment recurrence [33] and, to the best of our knowledge, the present study is the first to provide robust evidence that it is also associated with an increased risk of stromal microinvasion.

The American Society for Colposcopy and Cervical Pathology guidelines recommend that information on lesion size be included in the colposcopy report and used to tailor the excision accordingly [34,35]. By contrast, the size of CIN3 on the histopathologic sections has not yet been incorporated into the clinical decision-making. Our findings indicate that a given combination of lesion size and glandular crypt status is associated with a specific probability of stromal microinvasion.

The importance of this study lies both in its originality and in its large sample size. Measurements of the size of CIN3 are seldom reported in the histopathology reports of local excision specimens. At the St. Anna University Hospital, instead, this feature has been systematically recorded since the 1990s, when early studies suggested a possible association with the risk of disease persistence and recurrence [7,8]. Our experience illustrates that real-world observational studies can complement randomised controlled trials demonstrating the superior sensitivity of HPV testing for early CIN3 [1].

This study also has limitations that should be acknowledged. First, we had no information on patients’ HPV vaccination status and, if vaccinated, on the type of vaccine they had received. Two meta-analyses have shown that pre-surgical or adjuvant vaccination reduces the risk of developing recurrent HPV-related lesions [36,37].

Second, in addition to the screening test used, other aspects of clinical practice may have changed over the three decades covered by this study, including the histopathologic threshold for classifying a lesion as CIN3. To address this, we repeated the whole statistical strategy restricting to the years 2010–2018, a shorter time period during which HPV testing and cervical cytology were randomly assigned. The results were consistent with those obtained in the full dataset, indicating that temporal changes in clinical practice and selection factors, if present, did not introduce major biases.

Third, CIN3 diagnoses did not undergo central histologic review. However, a sample of CIN3 specimens from the Pathology Department involved in this study was independently and blindly reviewed during the New Technologies for Cervical Cancer (NTCC) screening trial [38], which was based in Turin. The observed level of agreement on the diagnosis of CIN3 (k = 0.57) was close to the boundary between moderate and substantial agreement [39]. Moreover, women enrolled in the NTCC trial were not invited to the organised screening programme, thereby avoiding another potential source of bias.

The clinical value of systematically recording the size of CIN3 warrants further investigation. Future studies should explore additional expected correlates of HPV test-detected CIN3, particularly a reduction in cervical cone volume and, consequently, reductions in the risk of disease persistence and in the rate of preterm birth in subsequent pregnancies [9,10]. At the same time, attention should be paid to the integration of lesion size measurement with emerging techniques to identify transforming CIN lesions—such as the high-risk HPV E6/E7 mRNA test [40] and the analysis of the methylation levels of CADM1, MAL and MIR-124 [41]. An important line of research will be to identify which genotypes are associated with the highest risk of lesion size progression, extensive glandular crypt involvement and stromal microinvasion.

## 5. Conclusions

In summary, we confirmed the hypothesis that the HPV test detects CIN3 lesions of smaller size than those detected by cervical cytology and, for the first time, we demonstrated that HPV test-detected CIN3 has a lower risk of stromal microinvasion. This association is mediated by the smaller lesion size and the lower degree of glandular crypt involvement, which interact in an additive manner. Other clinical correlates of HPV test-detected CIN3, including the impact on treatment outcomes and the obstetric sequelae, warrant further evaluation.

## Figures and Tables

**Table 1:** Association of detection mode with a lesion size (or linear extension) >6 mm and a massive glandular crypt involvement in CIN3.

Detection Mode	Total Number of Patients	Patients with a Lesion Size >6 mm				Patients with a Massive Glandular Crypt Involvement		
		Number (%)	Odds Ratio (95% CI) *	*p*		Number (%)	Odds Ratio (95% CI) *	*p*
Cervical cytology	2588	1299 (50.2)	1.00 (ref.)	<0.001		678 (26.2)	1.00 (ref.)	<0.001
HPV test	1156	455 (39.4)	0.67 (0.58–0.77)			167 (14.4)	0.47 (0.39–0.56)	

CIN3, cervical intraepithelial neoplasia grade 3; CI, confidence interval; ref., reference; HPV, human papillomavirus. *p* values are for the Wald test. * From a multiple logistic regression model adjusted for patient age.

**Table 2:** Association of detection mode with size/involvement (i.e., the composite variable combining the lesion size (or linear extension) and the degree of glandular crypt involvement) in CIN3.

Detection Mode	Total Number of Patients	Number (%) of Patients by Size/Involvement *					Odds Ratio (95% CI) †			
		≤6 mm,	>6 mm,	≤6 mm,	>6 mm,		≤6 mm,	>6 mm,	≤6 mm,	>6 mm,
		<Massive	<Massive	Massive	Massive		<Massive	<Massive	Massive	Massive
Cervical cytology	2588	1106 (42.7)	804 (31.1)	183 (7.1)	495 (19.1)		1.00 (ref.)	1.00 (ref.)	1.00 (ref.)	1.00 (ref.)
HPV test	1156	665 (57.5)	324 (28.0)	36 (3.1)	131 (11.3)		1.00 (ref.)	0.71 (0.60–0.83)	0.33 (0.22–0.47)	0.44 (0.35–0.55)

CIN3, cervical intraepithelial neoplasia grade 3; CI, confidence interval; ref., reference; HPV, human papillomavirus. * The value of 6 mm is the median lesion size; <massive indicates absent involvement or involvement of <50% of the gland depth; massive indicates involvement of ≥50% of the gland depth. † From a multinomial logistic regression model adjusted for patient age.

**Table 3:** Association of patient age and detection mode with the presence of stromal microinvasion in CIN3.

	Total Number of Patients	Patients with Stromal Microinvasion		
		Number (%)	Odds Ratio (95% CI) *	*p*
Patient age				0.250
30–39	1934	49 (2.5)	1.00 (ref.)	
40–49	1221	40 (3.3)	1.40 (0.91–2.14)	
50–59	466	16 (3.4)	1.59 (0.89–2.85)	
60–64	123	2 (1.6)	0.74 (0.18–3.07)	
Detection mode				0.004
Cervical cytology	2588	87 (3.4)	1.00 (ref.)	
HPV test	1156	20 (1.7)	0.48 (0.29–0.78)	

CIN3, cervical intraepithelial neoplasia grade 3; CI, confidence interval; ref., reference; HPV, human papillomavirus. Stromal microinvasion was defined as an invasion (measured from the base of the epithelium from which the carcinoma arises to the deepest invasive focus) ≤3 mm (microinvasive or stage IA1 cervical carcinoma according to the International Federation of Gynaecology and Obstetrics 2018 staging system) [21]. *p* values are for the Wald test. * From a multiple logistic regression model.

**Table 4:** Association of patient age, detection mode, and size/involvement (i.e., the composite variable combining the lesion size (or linear extension) and the degree of glandular crypt involvement) with the presence of stromal microinvasion in CIN3.

	Total Number of Patients	Patients with Stromal Microinvasion		
		Number (%)	Odds Ratio (95% CI) *	*p*
Patient age				0.327
30–39	1934	49 (2.5)	1.00 (ref.)	
40–49	1221	40 (3.3)	1.30 (0.84–2.00)	
50–59	466	16 (3.4)	1.64 (0.90–2.98)	
60–64	123	2 (1.6)	0.76 (0.18–3.26)	
Detection mode				0.148
Cervical cytology	2588	87 (3.4)	1.00 (ref.)	
HPV test	1156	20 (1.7)	0.69 (0.41–1.14)	
Size/involvement †				<0.001
≤6 mm, <massive	1771	8 (0.5)	1.00 (ref.)	
>6 mm, <massive	1128	25 (2.2)	4.93 (2.21–11.00)	
≤6, massive	219	12 (5.5)	11.91 (4.79–29.63)	
>6 mm, massive	626	62 (9.9)	23.07 (10.94–48.65)	

CIN3, cervical intraepithelial neoplasia grade 3; CI, confidence interval; ref., reference; HPV, human papillomavirus. Stromal microinvasion was defined as an invasion (measured from the base of the epithelium from which the carcinoma arises to the deepest invasive focus) ≤3 mm (microinvasive or stage IA1 cervical carcinoma according to the International Federation of Gynaecology and Obstetrics 2018 staging system) [21]. *p* values are for the Wald test. * From a multiple logistic regression model. † The value of 6 mm is the median lesion size; <massive indicates absent involvement or involvement of <50% of the gland depth; massive indicates involvement of ≥50% of the gland depth.

## Data Availability

All authors had full access to all of the data in this study and take complete responsibility for the integrity of the data and the accuracy of data analysis. The datasets generated and analysed during the current study are available from the corresponding author upon reasonable request.

## Supplementary Materials

The following supporting information can be downloaded at: https://www.mdpi.com/article/10.3390/cancers18030396/s1, Supplementary Figure S1: Pilot implementation study of the switch to human papillomavirus (HPV) testing in the organised cervical screening programme serving the metropolitan area of Turin (northern Italy) and some neighbouring districts: increasing ratio between CIN3 lesions detected by the HPV test and those detected by cervical cytology. Supplementary Table S1: Association of detection mode with a lesion size (or linear extension) >6 mm and a massive glandular crypt involvement in CIN3. The model shown in Table 1 of primary results was run on the restricted dataset for the years 2010–2018, when HPV testing and cervical cytology were randomly allocated. Supplementary Table S2: Association of detection mode with size/involvement (i.e., the composite variable combining the lesion size (or linear extension) and the degree of glandular crypt involvement) in CIN3. The model shown in Table 2 of primary results was run on the restricted dataset for the years 2010–2018, when HPV testing and cervical cytology were randomly allocated. Supplementary Table S3: Association of patient age and detection mode with the presence of stromal microinvasion in CIN3. The model shown in Table 3 of primary results was run on the restricted dataset for the years 2010–2018, when HPV testing and cervical cytology were randomly allocated. Supplementary Table S4: Association of patient age, detection mode, and size/involvement (i.e., the composite variable combining the lesion size (or linear extension) and the degree of glandular crypt involvement) with the presence of stromal microinvasion in CIN3. The model shown in Table 4 of primary results was run on the restricted dataset for the years 2010–2018, when HPV testing and cervical cytology were randomly allocated. Supplementary Table S5: Association of patient age, detection mode, and size/involvement (i.e., the composite variable combining the lesion size (or linear extension) and the degree of glandular crypt involvement) with the presence of stromal microinvasion in CIN3. For sensitivity analysis purposes, patients with CIN3 lesions with positive surgical margins, which were presumed to be incompletely excised and therefore to have an underestimated size, were excluded from the model.
