# Supplementary material for: Human Papillomavirus Testing and Size of CIN3: Implications for the Risk of Microinvasive Cervical Carcinoma
Source: Cancers (Basel). 2026 Jan 27;18(3):396. doi: 10.3390/cancers18030396 (PMC12896776; doi:10.3390/cancers18030396)
Supplement: Supplementary file 1 [file cancers-18-00396-s001.zip › cancers-3999795-supplementary.pdf]

cancers MDPI

Article

Human papillomavirus Testing and size of CIN3: Implications for the Risk of Microinvasive Cervical Carcinoma

Mario Preti [1], Annibale Biggeri [2], Guglielmo Ronco [3], Maria Kyrgiou [4,5], Raffaella Rizzolo [3], Paola Armaroli [6], Niccolò Gallio [1], Murat Gultekin [7], Federica Zamagni [8,*], Silvano Costa [9], Pedro Vieira-Baptista [10,11], Fulvio Borella [1], Stefano Cosma [1], Luigia Macrì [12], Christine Bergeron [13], Silvia Mancini [8], Laura De Marco [14], Daniele Tota [12] and Lauro Bucchi [8]

1 Department of Surgical Sciences, St. Anna University Hospital, Via Ventimiglia 3, 10126 Torino, Italy; mario.preti@unito.it (M.P.); niccolo.gallio@edu.unito.it (N.G.); fulvio.borella@unito.it (F.B.); stefano.cosma@unito.it (S.C.)
2 Unit of Biostatistics, Epidemiology and Public Health, Department of Cardiac, Thoracic, Vascular Sciences and Public Health, University of Padua, Via Leonardo Loredan 18, 35131 Padua, Italy; annibale.biggeri@ubep.unipd.it
3 Città della Salute e della Scienza University Hospital, Corso Bramante 88, 10126 Turin, Italy; guglielmo.ronco@cpo.it (G.R.); raffaella.rizzolo@cpo.it (R.R.)
4 Institute of Reproductive and Developmental Biology, Department of Metabolism, Digestion and Reproduction – Surgery and Cancer, Faculty of Medicine, Imperial College London, Du Cane Road, London W12 0NN, UK; m.kyrgiou@imperial.ac.uk
5 Imperial College Healthcare NHS Trust, Du Cane Road, London W12 0HS, UK
6 Epidemiology and Screening Unit, Reference Centre for Epidemiology and Cancer Prevention (CPO), Città della Salute e della Scienza University Hospital, Via Cavour 31, 10123 Turin, Italy; paola.armaroli@cpo.it
7 Division of Gynaecological Oncology, Department of Obstetrics and Gynaecology, Hacettepe University, Faculty of Medicine, Sihhiye, Ankara 06100, Türkiye; mrtgultekin@yahoo.com
8 Emilia-Romagna Cancer Registry, Romagna Cancer Institute, IRCCS Istituto Romagnolo per lo Studio dei Tumori (IRST) Dino Amadori, Via Piero Maroncelli 40, 47014 Meldola, Italy; silvia.mancini@irst.emr.it (S.M.); lauro.bucchi@irst.emr.it (L.B.)
9 Gynaecology Division, Villa Chiara Hospital, Via Porrettana 170, 40033 Casalecchio di Reno, Italy; costa.silvano@libero.it
10 Department of Gynecology-Obstetrics and Pediatrics, Faculdade de Medicina da Universidade do Porto, Alameda Professor Hernâni Monteiro, 4200-319 Porto, Portugal; pedrovieirabaptista@gmail.com
11 HPV and Vulvovaginal Pathology Unit, Hospital Lusíadas Porto, Av. da Boavista 171, 4050-115 Porto, Portugal
12 Pathology Unit, Città della Salute e della Scienza University Hospital, Corso Bramante 88, 10126 Turin, Italy; lmacri@cittadellasalute.to.it (L.M.); datota@cittadellasalute.to.it (D.T.)
13 CerbaPath, 30-32 Boulevard de Vaugirard, 75015 Paris, France; bergeron@lab-cerba.com
14 Cancer Epidemiology Unit, Reference Centre for Epidemiology and Cancer Prevention (CPO), Città della Salute e della Scienza University Hospital, Corso Bramante 88, 10126 Turin, Italy; laura.demarco@cpo.it
* Correspondence: federica.zamagni@irst.emr.it

Supplementary Materials

Table of contents

Supplementary Figure S1
Supplementary Table S1
Supplementary Table S2
Supplementary Table S3
Supplementary Table S4
Supplementary Table S5

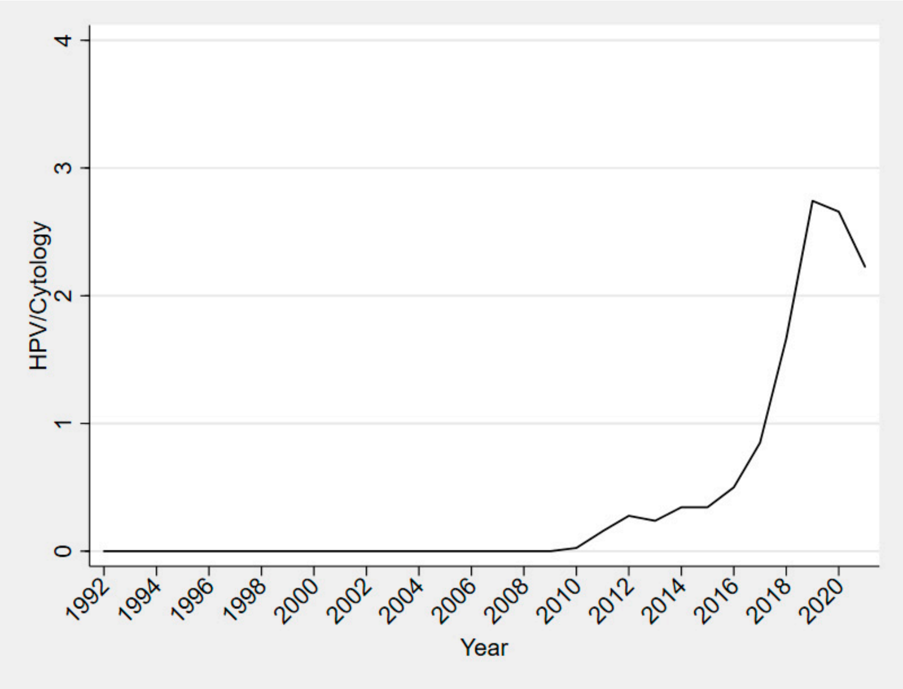

Supplementary Figure S1. Pilot implementation study of the switch to human papillomavirus (HPV) testing in the organised cervical screening programme serving the metropolitan area of Turin (northern Italy) and some neighbouring districts: increasing ratio between CIN3 lesions detected by the HPV test and those detected by cervical cytology.

Supplementary Table S1. Association of detection mode with a lesion size (or linear extension) >6 mm and a massive glandular crypt involvement in CIN3. The model shown in Table 1 of primary results was run on the restricted dataset for the years 2010-2018, when HPV testing and cervical cytology were randomly allocated.

Detection Mode	Total Number of Patients	Patients with a Lesion Size >6 mm			Patients with a Massive Glandular Crypt Involvement		
		Number (%)	Odds Ratio (95% CI)*	*p*	Number (%)	Odds Ratio (95% CI)*	*p*
Cervical cytology	1261	613 (48.6)	1.00 (ref.)	0.054	335 (26.6)	1.00 (ref.)	<0.001
HPV test	661	283 (42.8)	0.83 (0.68-1.00)		126 (19.1)	0.64 (0.50-0.81)	

CIN3, cervical intraepithelial neoplasia grade 3; CI, confidence interval; ref., reference; HPV, human papillomavirus. *p* values are for the Wald test. *From a multiple logistic regression model adjusted for patient age.

Supplementary Table S2. Association of detection mode with size/involvement (i.e., the composite variable combining the lesion size (or linear extension) and the degree of glandular crypt involvement) in CIN3. The model shown in Table 2 of primary results was run on the restricted dataset for the years 2010-2018, when HPV testing and cervical cytology were randomly allocated.

Detection Mode	Total Number of Patients	Number (%) of Patients by Size/Involvement*				Odds Ratio (95% CI) †			
		≤6 mm, <Massive	>6 mm, <Massive	≤6 mm, Massive	>6 mm, Massive	≤6 mm, <Massive	>6 mm, <Massive	≤6 mm, Massive	>6 mm, Massive
Cervical cytology	1261	554 (43.9)	372 (29.5)	94 (7.5)	241 (19.1)	1.00 (ref.)	1.00 (ref.)	1.00 (ref.)	1.00 (ref.)
HPV test	661	351 (53.1)	184 (27.8)	27 (4.1)	99 (15.0)	1.00 (ref.)	0.84 (0.67-1.06)	0.47 (0.29-0.74)	0.65 (0.49-0.85)

CIN3, cervical intraepithelial neoplasia grade 3; CI, confidence interval; ref., reference; HPV, human papillomavirus. * The value of 6 mm is the median lesion size; <massive indicates absent involvement or involvement of <50% of the gland depth; massive indicates involvement of ≥50% of the gland depth. † From a multinomial logistic regression model adjusted for patient age.

Supplementary Table S3. Association of patient age and detection mode with the presence of stromal microinvasion in CIN3. The model shown in Table 3 of primary results was run on the restricted dataset for the years 2010-2018, when HPV testing and cervical cytology were randomly allocated.

	Total Number of Patients	Patients with Stromal Microinvasion		
		Number (%)	Odds Ratio (95% CI)*	*p*
Patient age†	1922	52 (2.7)	1.13 (0.80-1.60)	0.475
30-39				
40-49				
50-59				
60-64				
Detection mode				0.018
Cervical cytology	1261	42 (3.3)	1.00 (ref.)	
HPV test	661	10 (1.5)	0.43 (0.21-0.86)	

CIN3, cervical intraepithelial neoplasia grade 3; CI, confidence interval; ref., reference; HPV, human papillomavirus. Stromal microinvasion was defined as an invasion (measured from the base of the epithelium from which the carcinoma arises to the deepest invasive focus) ≤3 mm (microinvasive or stage IA1 cervical carcinoma according to the International Federation of Gynaecology and Obstetrics 2018 staging system) [21]. *p* values are for the Wald test. * From a multiple logistic regression model. † Patient age was entered in the model as a linear variable.

Supplementary Table S4. Association of patient age, detection mode, and size/involvement (i.e. the composite variable combining the lesion size (or linear extension) and the degree of glandular crypt involvement) with the presence of stromal microinvasion in CIN3. The model shown in Table 4 of primary results was run on the restricted dataset for the years 2010-2018, when HPV testing and cervical cytology were randomly allocated.

	**Total Number of Patients**	**Patients with Stromal Microinvasion**		
		**Number (%)**	**Odds Ratio (95% CI)***	***p***
Patient age†	1922	52 (2.7)	1.14 (0.79-1.64)	0.482
30-39				
40-49				
50-59				
60-64				
Detection mode				0.069
Cervical cytology	1261	42 (3.3)	1.00 (ref.)	
HPV test	661	10 (1.5)	0.51 (0.25-1.05)	
Size/involvement‡				<0.001
≤6 mm, <massive	905	3 (0.3)	1.00 (ref.)	
>6 mm, <massive	556	11 (2.0)	6.00 (1.66-21.63)	
≤6, massive	121	5 (4.1)	11.93 (2.81-50.75)	
>6 mm, massive	340	33 (9.7)	30.91 (9.4-101.63)	

CIN3, cervical intraepithelial neoplasia grade 3; CI, confidence interval; ref., reference; HPV, human papillomavirus. Stromal microinvasion was defined as an invasion (measured from the base of the epithelium from which the carcinoma arises to the deepest focus of invasion) ≤3 mm (microinvasive or stage IA1 cervical carcinoma according to the International Federation of Gynaecology and Obstetrics 2018 staging system) [21]. *p* values are for the Wald test. * From a multiple logistic regression model. † Patient age was entered in the model as a linear variable. ‡ The value of 6 mm is the median lesion size; <massive indicates absent involvement or involvement of <50% of the gland depth; massive indicates involvement of ≥50% of the gland depth.

Supplementary Table S5. Association of patient age, detection mode, and size/involvement (i.e. the composite variable combining the lesion size (or linear extension) and the degree of glandular crypt involvement) with the presence of stromal microinvasion in CIN3. For sensitivity analysis purposes, patients with CIN3 lesions with positive surgical margins, which were presumed to be incompletely excised and therefore to have an underestimated size, were excluded from the model.

	Total Number of Patients	Patients with Stromal Microinvasion		
		Number (%)	Odds Ratio (95% CI)*	*p*
Patient age				0.968
30-39	849	16 (1.9)	1.00 (ref.)	
40-49	504	10 (2.0)	0.99 (0.44-2.26)	
50-59	182	2 (1.1)	0.69 (0.15-3.14)	
60-64	48	1 (2.1)	1.10 (0.13-9.04)	
Detection mode				0.298
Cervical cytology	1058	20 (1.9)	1.00 (ref.)	
HPV test	525	9 (1.7)	1.57 (0.67-3.68)	
Size/involvement†				<0.001
≤6 mm, <massive	931	5 (0.5)	1.00 (ref.)	
>6 mm, <massive	440	6 (1.4)	2.67 (0.80-8.87)	
≤6, massive	53	4 (7.5)	17.56 (4.36-70.66)	
>6 mm, massive	159	14 (8.8)	19.25 (6.70-55.35)	

CIN3, cervical intraepithelial neoplasia grade 3; CI, confidence interval; ref., reference; HPV, human papillomavirus. Stromal microinvasion was defined as an invasion (measured from the base of the epithelium from which the carcinoma arises to the deepest focus of invasion) ≤3 mm (microinvasive or stage IA1 cervical carcinoma according to the International Federation of Gynaecology and Obstetrics 2018 staging system) [21]. *p* values are for the Wald test. * From a multiple logistic regression model. † The value of 6 mm is the median lesion size; <massive indicates absent involvement or involvement of <50% of the gland depth; massive indicates involvement of ≥50% of the gland depth.
